# DNA methylation-regulated SNX20 overexpression correlates with poor prognosis, immune cell infiltration, and low-grade glioma progression

**DOI:** 10.18632/aging.204144

**Published:** 2022-06-27

**Authors:** Xi Chen, Xiulin Jiang, Heping Wang, Chunyan Wang, Chenyang Wang, Chenglong Pan, Fan Zhou, Jintao Tian, Xiaoqun Niu, Zhi Nie, Wei Chen, Xiaobin Huang, Jun Pu, Chen Li

**Affiliations:** 1 Department of Neurosurgery, The second Affiliated Hospital of Kunming Medical University, Kunming 650223, China; 2 Key Laboratory of Animal Models and Human Disease Mechanisms of Chinese Academy of Sciences and Yunnan Province, Kunming Institute of Zoology, Kunming 650223, Yunnan, China; 3 Department of Pathology, First Affiliated Hospital of Kunming Medical University, Kunming 650032, Yunnan, China; 4 Hematology and Rheumatology Department, The Pu’er People’s Hospital, Pu’er 665000, China; 5 Department of Respiratory Medicine, The 2nd Affiliated Hospital of Kunming Medical University, Kunming, Yunnan Province, China; 6 Department of Neurology, First Affiliated Hospital of Kunming Medical University, Kunming 650032, Yunnan, China; 7 Department of Biology, Chemistry, Pharmacy, Free University of Berlin, Berlin 14195, Germany

**Keywords:** gliomas, SNX20, DNA methylation, prognostic biomarker, immune infiltration

## Abstract

We revealed that SNX20 was up-regulated in LGG, and its higher expression was associated with adverse clinical outcomes and poor clinical characteristics, including WHO grade, IDH mutation, 1p/19q codeletion, and primary therapy outcome. The results of the Cox regression analysis revealed that SNX20 was an independent factor for the prognosis of low-grade glioma. Meanwhile, we also established a nomogram based on SNX20 to predict the 1-, 3-, or 5-year survival in LGG patients. Furthermore, we found that DNA hypomethylation results in its overexpression in LGG. In addition, functional annotation confirmed that SNX20 was mainly involved in the immune response and inflammatory response related signaling pathways, including the T cell receptor signaling pathway, natural killer cell-mediated cytotoxicity, and the NF-kappa B signaling pathway. Finally, we determined that increased expression of SNX20 was correlated with infiltration levels of various immune cells and immune checkpoint in LGG. Importantly, we found that SNX20 was highly expressed in glioma cell lines. Depletion of SNX20 significantly inhibits glioma cell proliferation and migration abilities. This is the first study to identify SNX20 as a new potential prognostic biomarker and characterize the functional roles of SNX20 in the progression of LGG, and provides a novel potential diagnostic and therapeutic biomarker for LGG in the future.

## INTRODUCTION

Glioma is a kind of brain tumor originated from the central nervous system. Its mortality and recurrence rate are very high [1]. Despite different treatment and diagnosis methods, the survival rate of glioma patients is still disappointing. Therefore, identified novel and specific diagnostic markers is very important to improve the survival rate and prognosis of glioma patients.

SNX20, as a member of Sorting nexins proteins family, and plays an indispensable role in protein sorting and transport [2]. Increased expression of SNX20 has been shown to predict clinical therapy response to PD-1inhibitors of NSCLC [3]. Furthermore, elevated expression of SNX20 promotes the redistribution of PSGL-1 from the cell surface to endosomal organelles [4]. Our previous study found that SNX20 decreased in non-small cell lung cancers (NSCLC). Overexpression of SNX20 prominently inhibits NSCLC cell proliferation and migration abilities. Above results show that SNX20 functions as a tumor suppressor in non-small cell lung cancer. However, it remains unclear the biological function and immune infiltration role of SNX20 in glioma progression.

In this study, we found that SNX20 was highly expressed in glioma and correlated with unfavourable clinical outcomes. Cox regression analysis confirmed that SNX20 was an independent prognosis factor for glioma prognosis. Meanwhile, we also established a nomogram using SNX20 to predict the overall survival time in LGG patients. Furthermore, we found that DNA hypo-methylation results in its overexpression in LGG. In addition, the functional annotation confirms that SNX20 is major participate in the immune response and the inflammatory response-related signaling pathway, including the cytotoxicity mediated by natural killer cells. Finally, we uncover that higher expression of SNX20 was related to the infiltration levels of various immune cells and immune checkpoint in LGG. More importantly, we uncover that SNX20 was up-regulated in GBM cell lines. Depletion of SNX20 evidently inhibited glioma cell growth and migration abilities. This study firstly uncover that the potential biological functional of SNX20 in the progression of LGG, which represents a potential diagnostic and prognostic biomarker for glioma in the future.

## MATERIALS AND METHODS

### Data download

We download the transcriptome sequencing data and clinical information of glioma patients from the TCGA (https://www.cancer.gov/tcga). We also used CGGA (http://www.cgga.org.cn/analyse/RNA-data.jsp) dataset to validate the expression, clinical relevance and prognosis values of SNX20 in glioma.

### Analysis of the correlation between DNA methylation and SNX20 expression

We utilized MethSurv (https://biit.cs.ut.ee/methsurv/) and SMART (http://www.bioinfo-zs.com/smartapp/) to determine the relationship between DNA methylation and SNX20 expression, prognosis of glioma patients [5, 6].

### GO and KEGG analysis of SNX2

In this finding, we employed the linkedomics to gain the co-expression genes of SNX20 in glioma, and utilized clusterProfiler package perform the GO and KEGG enrichment of SNX20 in LGG [7–9].

### Immune infiltration analysis

In this finding, we employed to the TIMER (https://cistrome.shinyapps.io/timer/) database to examine the potential immune role of SNX20 in glioma [10].

### Cell culture conditions and siRNA interference

GBM cells lines were purchased from cell bank of Kunming Institute of Zoology, and cultured in DMEM medium supplemented with 10% fetal bovine serum (FBS) and 1% penicillin/streptomycin at 37° C in atmosphere containing 95% air and 5% CO2.

### QPCR assay

The qRT-PCR assay was performed as documented [11]. The primer sequences are list follows SNX20-F: ACCTGACGGGCACTTAGACA, SNX20-R: AGAGCAGTTTGACGTGCTTCC; β-actin-F: CTTCGCGGGCGACGAT, β-actin-R: CCATAGGAATCCTTCTGACC. The expression quantification was obtained with the 2−ΔΔCt method.

### Biological function assay and statistical analysis

Cell proliferation and cell proliferation assay was conducted by previously described [11]. Correlation analysis was conducted by Pearson correlation test. P < 0.05 (*), P < 0.01 (**) and P < 0.001 (***), were considered significant.

## RESULTS

### SNX20 was up-regulated in LGG

**Figure 1:**
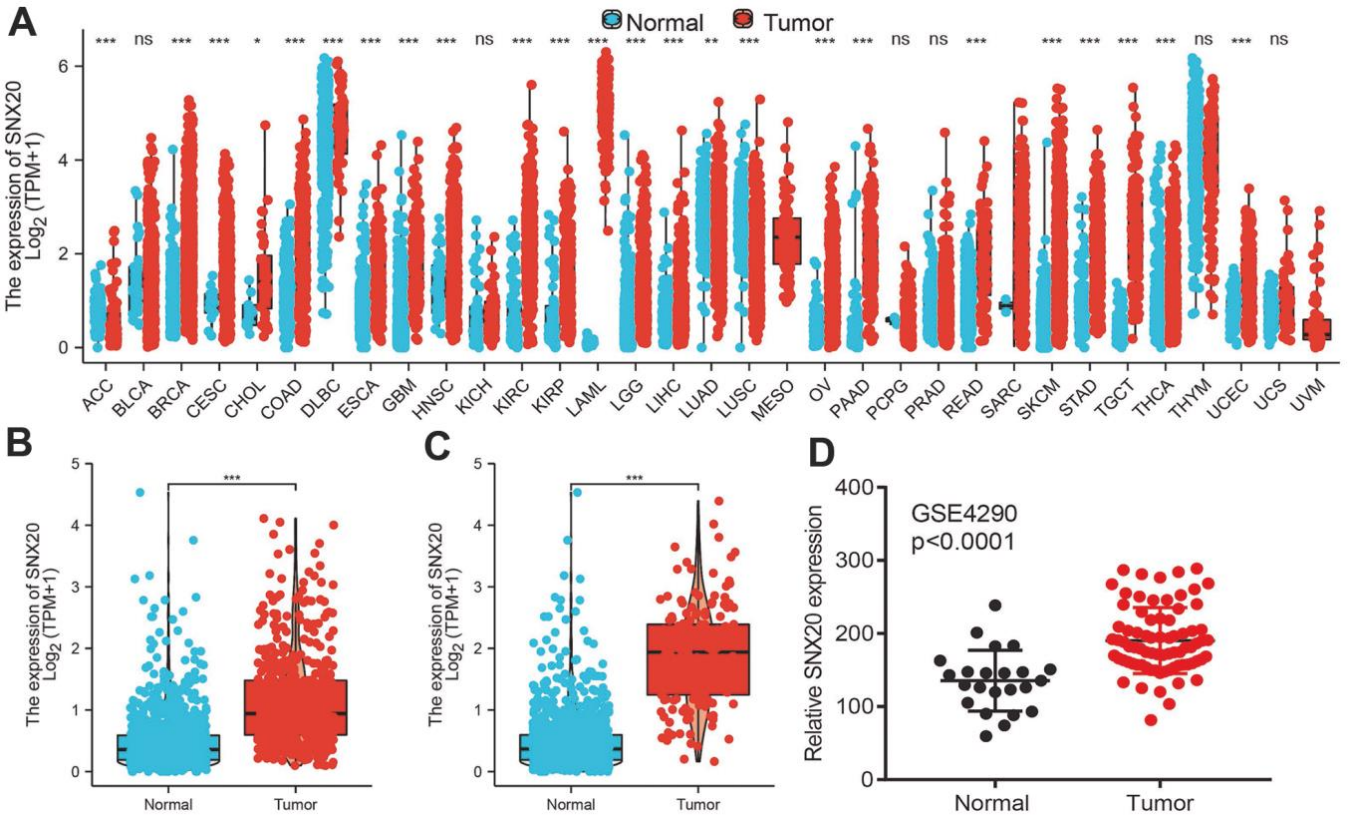
**SNX20 is highly expressed in LGG.** (**A**) The expression of SNX20 in pan-cancer examined by TCGA database. (**B**–**D**) The expression of SNX20 in LGG examine by TCGA/GEO databases.

We used the TCGA and the Genotype-Tissue Expression (GTEx) databases to explore the expression of SNX20 in various cancers, Results showed that SNX20 differentially expressed in various human cancers (Figure 1A). Furthermore, we determined that SNX20 was increased in glioma tissue compared to the control group based on TCGA and GEO datasets (Figure 1B–1D). Results show that SNX20 was overexpressed in LGG tissue compared to controls.

### DNA methylation modulates SNX20 expression in LGG

**Figure 2:**
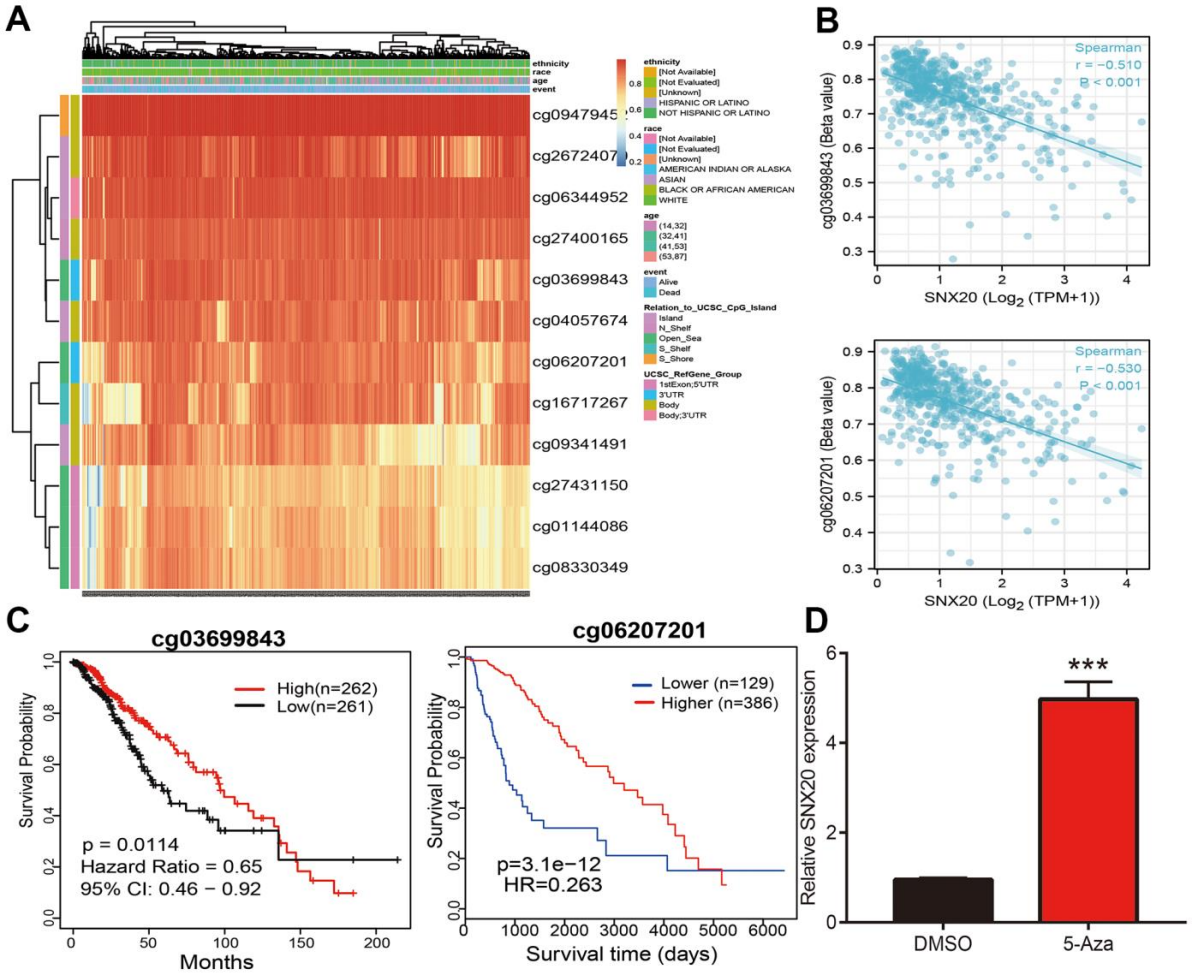
**Analysis of the DNA methylation level of SNX20.** (**A**) The DNA methylation sites of SNX20 in LGG. (**B**) The correlation between DNA methylation and SNX20 expression in LGG. (**C**) The prognosis for methylation level of SNX20 in TCGA dataset. (**D**) The expression of SNX20 in U87 cells after 5-Aza treatment examine by qRT-PCR assay.

First, we confirmed that there are various methylation sites in the promoter region of SNX20 (Figure 2A). Furthermore, the methylation sites (cg03699843 and cg06207201) were negatively related to the expression of SNX20 (Figure 2B). More importantly, using the methsurv statistical tool, we found that the decreased levels of methylation in cg03699843 and cg06207201 were related to adverse clinical outcomes in glioma patients (Figure 2C). Finally, to verify above hypothesis, we treated LGG cells with 5-azacytidine, an inhibitor of DNA methyltransferase [12], which cause increased SNX20 RNA levels in U87 cells (Figure 2D).

### Relationship between SNX20 expression and LGG clinical characteristics

**Figure 3:**
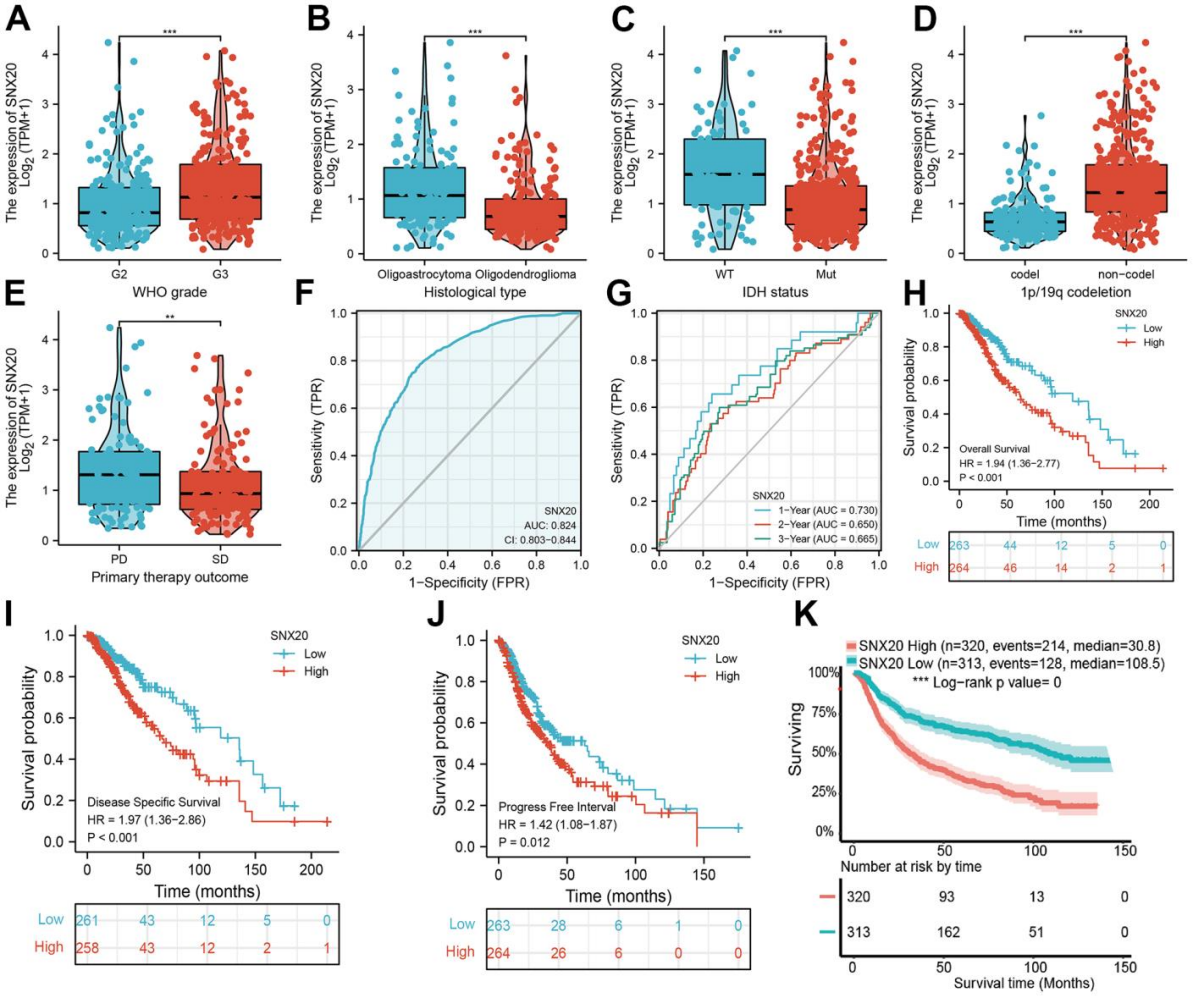
**The correlation between SNX20 expression and clinical information in LGG.** (**A**–**E**) The correlation between SNX20 expression and clinical features, including the higher tumor grades, histological type, IDH mutation status, 1p/19q chromosome co-deletion and primary therapy outcome. (**F**, **G**) ROC analyses revealed the predictive value of SNX20 in glioma based on TCGA-LGG. (**H**–**K**) The overall survival, disease specific survival and progression free survival of SNX20 in LGG examined by TCGA and CGGA database. Primary therapy outcome: including PD: progressive disease. SD: stable disease.

**Figure 4:**
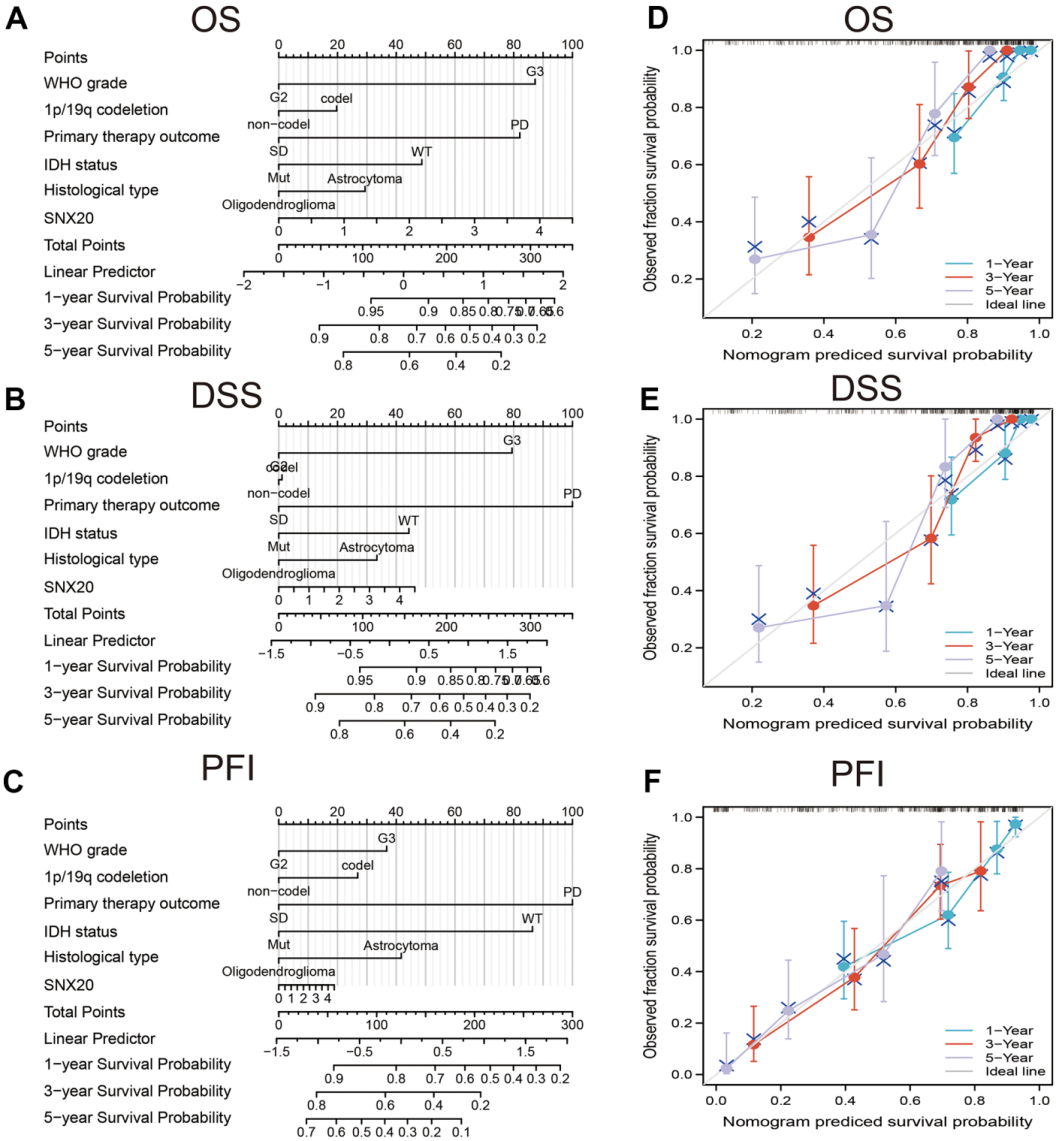
**Construction nomogram of predicted prognosis of SNX20 in LGG.** Construction nomogram to predicted (**A**) OS, (**B**) DSS, and (**C**) PFI in LGG patients. The calibration curve used to display the TCGA-LGG cohort for (**D**) OS, (**E**) DSS and (**F**) PFI.

We show that increased SNX20 expression was associated with adverse clinical characteristics (Figure 3A–3E). ROC curve results confirmed that AUC values of SNX20 were 0.824 in the TCGA-LGG dataset (Figure 3F). A time-dependent ROC curve was utilized to evaluate the predictive efficiency of SNX20 in predicting 1-, 3-, and 5-year overall survival, and the AUC value of the overall survival rate of patients with glioma was 0.730, 0.650, and 0.665, respectively (Figure 3G). We also found that up-regulation of SNX20 was associated with adverse clinical outcomes, including adverse OS, DSS, and PFS (Figure 3H–3J). This result was verified by the CGGA data set (Figure 3K). We used the SNX20 expression build a nomogram and used to predict overall survival, disease-specific survival, and the progression-free interval and to calculate the respective C-indexes 0.798(0.771–0.825), 0.811(0.785–0.837), and 0.767(0.745–0.789) (Figure 4A–4F).

### GO and KEGG analysis of SNX20

**Figure 5:**
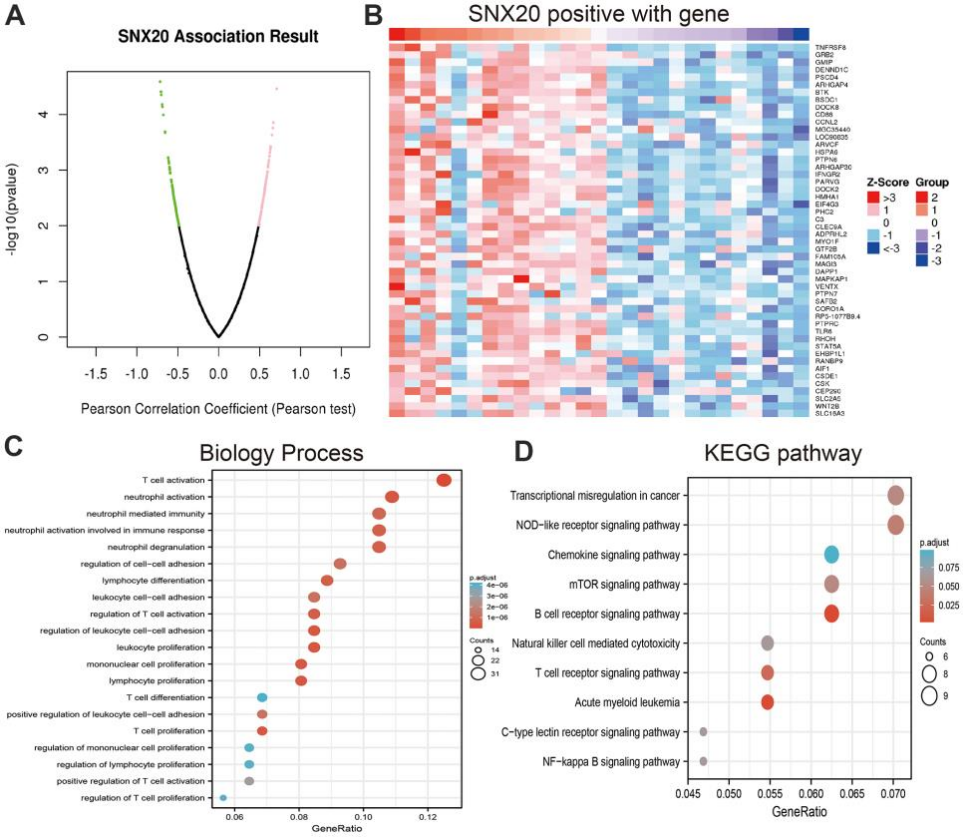
**Analysis the function of SNX20 expression in LGG.** (**A**, **B**) Analysis the co-expression genes of SNX20 in LGG examined by linkomics databases. (**C**) Analysis the biology process involved by SNX20 in LGG. (**D**) Analysis the KEGG signaling pathway of SNX20 in LGG.

To examine the biological functions of SNX20, using linkedomics tools, we obtained co-expressed genes that were positively related to SNX20 (Figure 5A, 5B). Furthermore, functional annotation showed that SNX20 was involved primarily in T cell proliferation, regulation of leukocyte cell–cell adhesion, and regulation of T cell proliferation, and T cell differentiation among the Gene Ontology (GO) annotation terms (Figure 5C). SNX20 participates mainly in the acute myeloid leukemia, NOD-like receptor signaling pathway, the mTOR signaling pathway (Figure 5D).

### GSEA enrichment of SNX20-related signaling pathways

**Figure 6:**
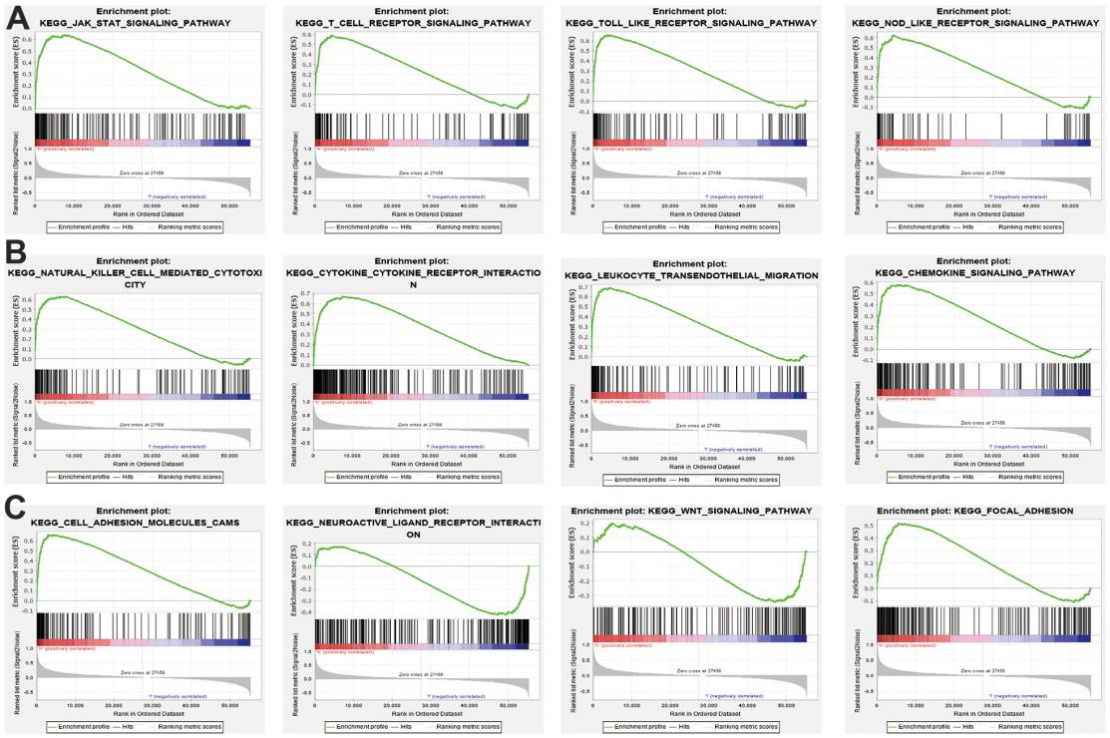
**KEGG signaling pathway explore by GSEA software.** (**A**–**C**) The signaling pathway involved by SNX20 in LGG examined by GSEA software.

To further explore the molecular mechanisms of SNX20 involvement in LGG, we conducted GSEA enrichment and found that high expression of SNX20 was major participated in the JAK/STAT signaling pathway, cytotoxicity mediated by natural killer cells, cytokine receptor interactions, transendothelial migration of leukocytes, the chemokine signaling pathway and in focal adhesion (Figure 6A–6C). Collectively, these findings confirmed that SNX20 plays an indispensable role in the regulation of immune responses. Therefore, targeting SNX20 may be an alternative strategy for cancer therapy.

### Immune cell infiltration analysis of SNX20

**Figure 7:**
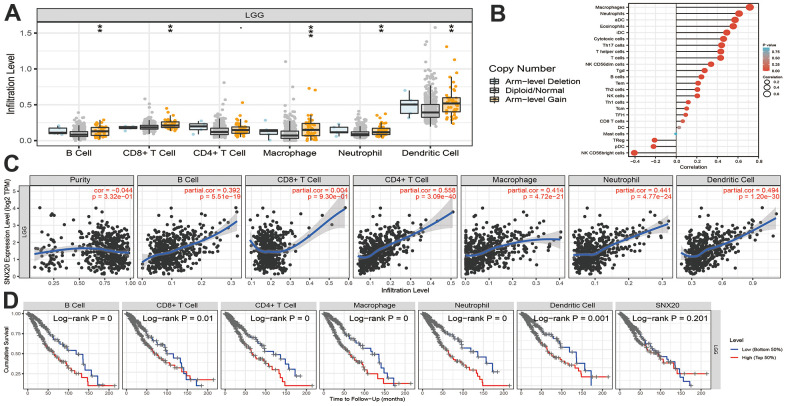
**Analysis the correlation between SNX20 expression and diverse immune cell infiltration.** (**A**) The correlation between SNX20 expression and somatic copy number alterations examine by TIMER. (**B**) The correlation between SNX20 expression and diverse immune cell infiltration. (**C**) The correlation between SNX20 expression and the infiltration levels of B cells, CD4+ T cells, CD8+ T cells, dendritic cells, Macrophages and Neutrophils. (**D**) The B cells, CD4+ T cells, CD8+ T cells, dendritic cells, Macrophages and Neutrophils are correlated with the cumulative survival rate in LGG examine by TIMER.

**Figure 8:**
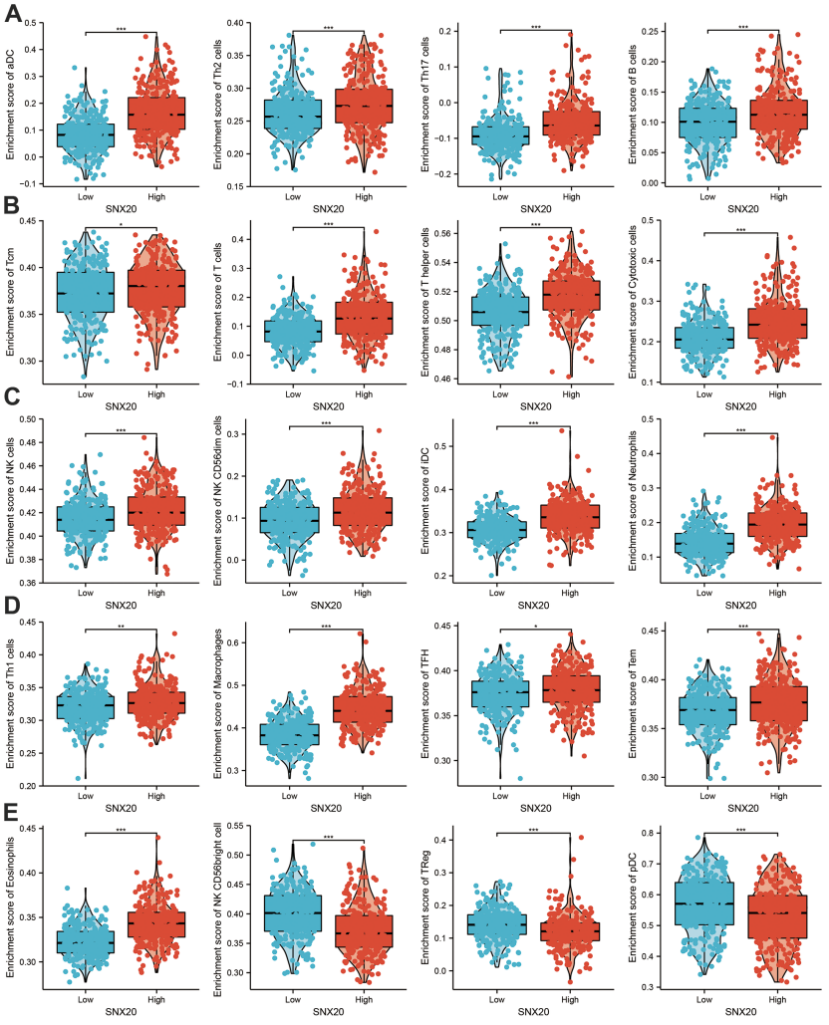
**Analysis the correlation between SNX20 expression and diverse immune cell infiltration.** (**A**–**E**) Diverse proportions of immune cell subtype in tumor samples in high and low SNX20 expression groups.

**Figure 9:**
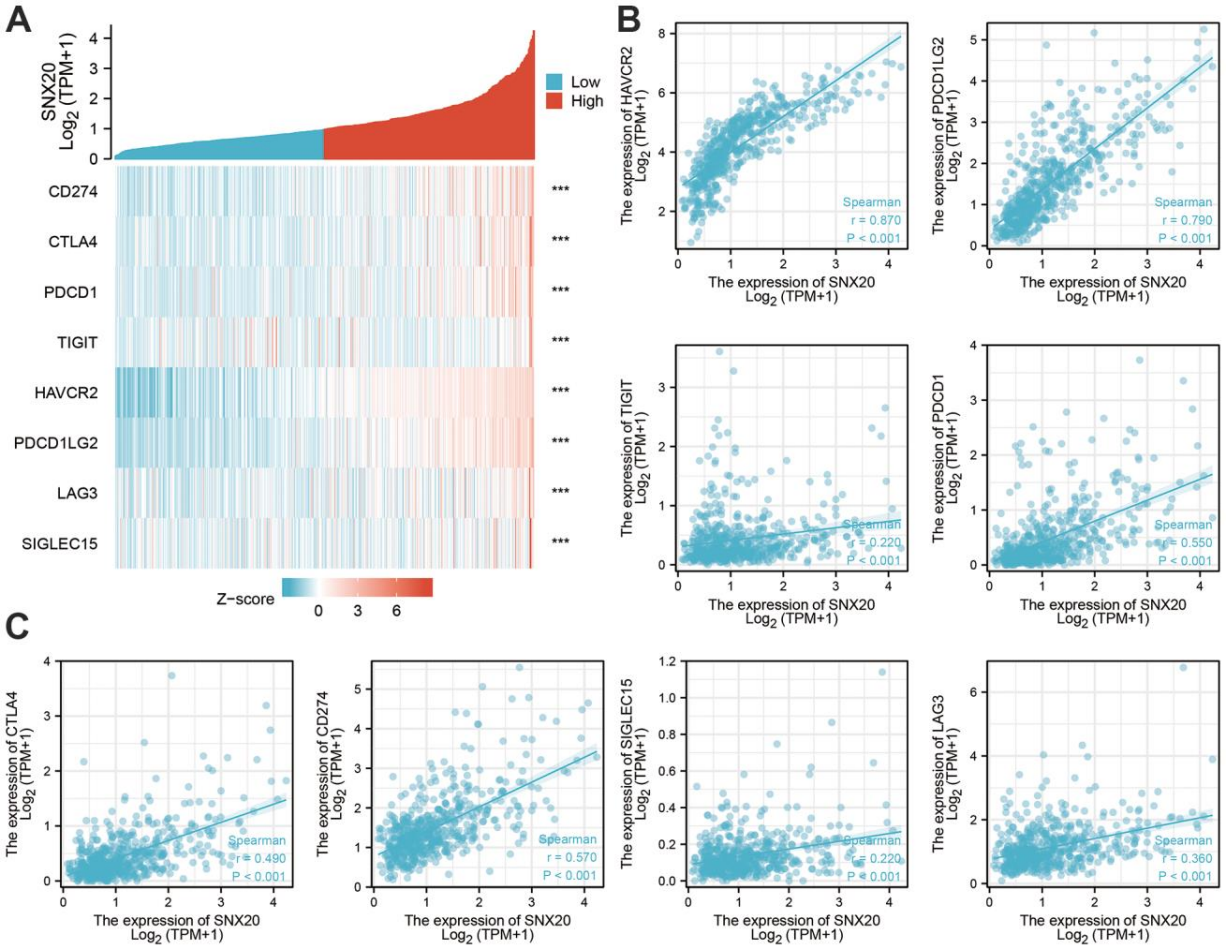
**The correlation between SNX20 expression and immune modulator in LGG.** (**A**–**C**) The correlation between the SNX20 expression and various immune checkpoints related genes.

First, we found that SNX20 somatic copy number significantly affected the infiltration levels of B cells and dendritic cells (Figure 7A). Furthermore, we show that SNX20 was positively related to the level of B cells, CD8+ T cells, and CD4+ T cells (Figure 7B, 7C). Our analysis using the Cox proportional hazard model demonstrated that SNX20 were significantly was related to poor overall survival in LGG patients (Figure 7D). Furthermore, we used ssGSEA to quantify the level of immune cell infiltration in the high- and low-expression groups of SNX20. We determined that increased expression of SNX20 was positively related to the abundance of 20 immune cells (Figure 8A–8E). Finally, we show that SNX20 expression was positively associated with the immune modulator in glioma (Figure 9A–9C).

### SNX20 knockdown inhibited GBM cell proliferation and migration

**Figure 10:**
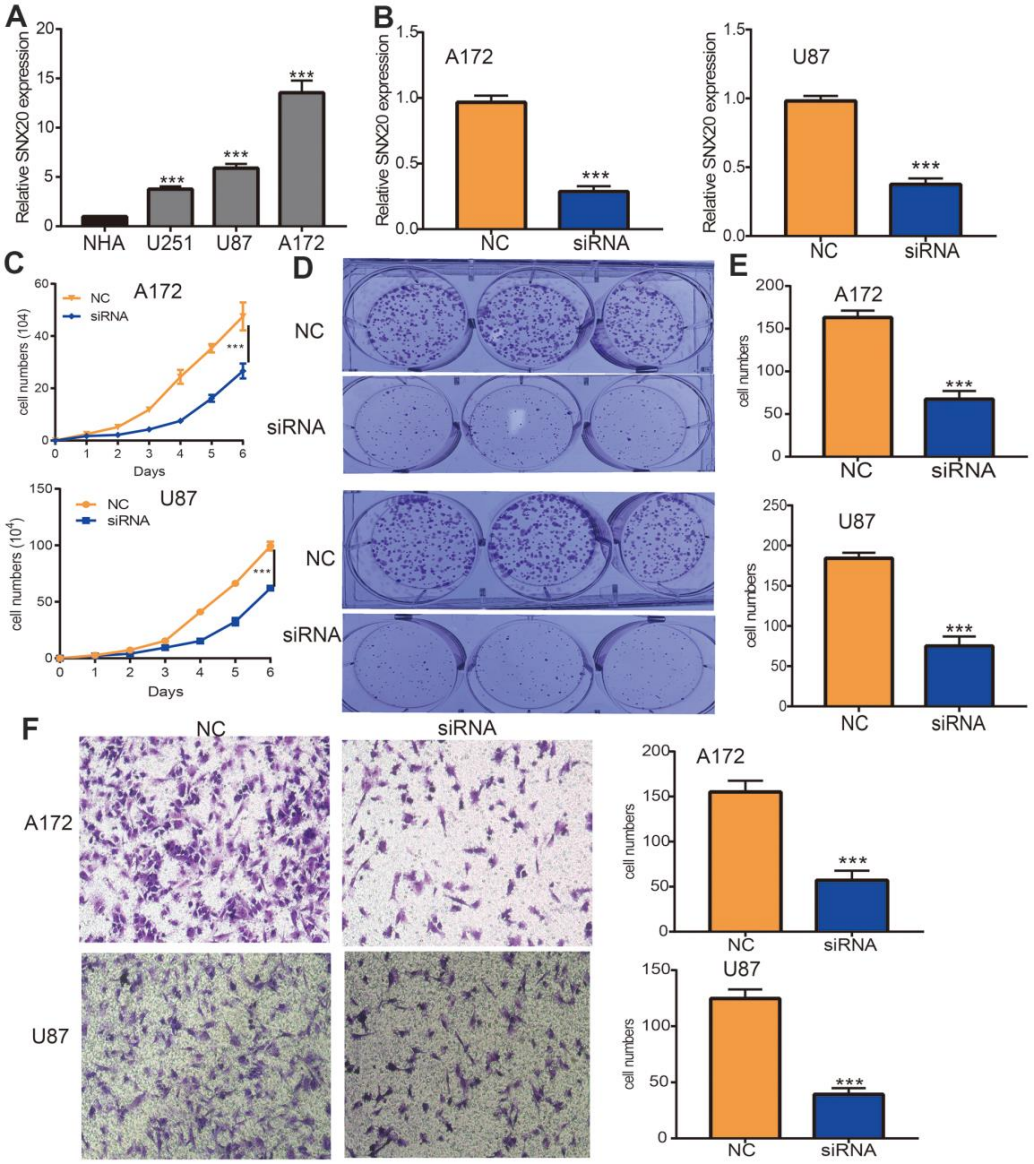
**Depletion of SNX20 inhibits GBM cell proliferation and migration.** (**A**) The expression of SNX20 in normal human astrocytes cells (NHA) and GBM cell lines (U87, A172 and U251). (**B**) The SNX20 knockdown efficiency in A172 and U87 cells were verified by qRT-PCR assay. (**C**–**E**) SNX20 knockdown significantly inhibited A172 and U87 cells proliferation examined by growth curve and colony formation assays. (**F**) SNX20 knockdown significantly inhibited A172 and U87 cells migration examined by transwell assay. Scale bar=50 μm. NC=Negative control, siRNA= SNX20 siRNA, * P < 0.05, ** P < 0.01, *** P < 0.001.

First, we found that SNX20 was elevated in GBM cell lines, especially A172 cells (Figure 10A). The qRT-PCR assay showed that SNX20 mRNA was reduced in U87 and A172 cells after treatment with targeted siRNA (Figure 10B). Second, we confirmed that SNX20 depletion significantly inhibits the proliferative ability of GBM cells (Figure 10C–10E). Furthermore, we discovered that SNX20 suppression reduced the migration ability of GBM cells examined by the Transwell assay (Figure 10F).

## DISCUSSION

SNX20, as a member of the Sorting nexins proteins family, plays an indispensable role in protein sorting and transport. In this study, we determined that SNX20 was up-regulated in the diverse human cancers, especially in glioma. Meanwhile, increased expression of SNX20 was associated with adverse clinical characteristics. Finally, we found that higher expression of SNX20 was correlated with poor clinical outcome, including overall survival, disease-specific survival, and progression-free survival. We also found that up-regulation of SNX20 was associated with adverse clinical outcomes, including adverse OS, DSS, and PFS. This result was verified by the CGGA dataset. We used the SNX20 expression build a nomogram and this nomogram used to predict overall survival, disease-specific survival, and the progression-free interval.

Accumulated research shows that that DNA methylation plays crucial role in gene expression regulation [13]. In this study, we confirmed that there are various methylation sites in the promoter region of SNX20. Furthermore, the methylation sites (cg03699843 and cg06207201) were negatively related to the expression of SNX20. More importantly, using the methsurv statistical tool, we found that the decreased levels of methylation in cg03699843 and cg06207201 were related to adverse clinical outcomes in glioma patients. Finally, to verify above hypothesis, we treated LGG cells with 5-azacytidine, an inhibitor of DNA methyltransferase [12], which cause increased SNX20 RNA levels in U87 cells.

Previous studies reported that SNX20 is necessary for the classification and trafficking of intracellular proteins [4]. In our finding, we showed that SNX20 was involved primarily in T cell proliferation, regulation of leukocyte cell–cell adhesion, and regulation of T cell proliferation, and T cell differentiation. GSEA enrichment and found that higher expression of SNX20 was major participated in the JAK/STAT signaling pathway, cytotoxicity mediated by natural killer cells, cytokine receptor interactions, the transendothelial migration of leukocytes, the chemokine signaling pathway and in focal adhesion.

Previous reports have suggested that SNX20 expression is strongly correlated with immune infiltration levels in lung adenocarcinoma [3]. In our study, we found that SNX20 was positively related to the level of B cells, CD8+ T cells, and CD4+ T cells. Our analysis using the Cox proportional hazard model demonstrated that SNX20 were significantly was related to poor overall survival in LGG patients. We determined that increased expression of SNX20 was positively related to the abundance of 20 immune cells.

Our previous work showed that SNX20 was down-regulated in NSCLC, overexpression of SNX20 inhibited cell growth and migration abilities of NSCLC cells [14]. However, no studies have reported the functions of SNX20 in LGG. In this study, we revealed that SNX20 was elevated in GBM cell lines, and depletion of SNX20 significantly inhibited GBM cell proliferation and migration abilities.

## CONCLUSIONS

Our findings confirmed that DNA hypomethylation-induced increased expression of SNX20 in LGG. Furthermore, up-regulation of SNX20 was positively related to immune cell infiltration and immune checkpoints. Finally, depletion of SNX20 significantly inhibited GBM cell proliferation and migration. SNX20 could be a novel potential target for diagnosis and treatment of glioma.
